# Patient and public involvement: Two sides of the same coin or different coins altogether?

**DOI:** 10.1111/bioe.12584

**Published:** 2019-04-08

**Authors:** Matthew S. McCoy, Jonathan Warsh, Leah Rand, Michael Parker, Mark Sheehan

**Affiliations:** ^1^ Department of Medical Ethics and Health Policy Perelman School of Medicine University of Pennsylvania PA USA; ^2^ The Ethox Centre Medical Sciences Division University of Oxford Oxford UK

**Keywords:** patient involvement, public engagement, public involvement

## Abstract

Patient and public involvement (PPI) has gained widespread support in health research and health policy circles, but there is little consensus on the precise meaning or justifications of PPI. We argue that an important step towards clarifying the meaning and justification for PPI is to split apart the familiar acronym and draw a distinction between patient and public involvement. Specifically, we argue that patient involvement should refer to the practice of involving individuals in health research or policy on the basis of their experience with a particular condition, while public involvement should refer to the practice of involving individuals in health policy or research based on their status as members of a relevant population. Analyzing cases from the UK, Australia, and the USA, we show how our proposed distinction can deliver much needed clarity to conversations on PPI, while guiding the development and evaluation of future PPI‐based policies.

## 1 INTRODUCTION

Over the last 20 years patient and public involvement (PPI) has gained widespread support in applied health research and health policy circles. Many funding bodies throughout Europe and North America now demand explicit evidence that grantees are pursuing PPI, while others provide strong encouragement to do so. In the UK the *Health and Social Care Act of 2001* made it a legal requirement for hospitals, health‐care providers, and research institutions to involve members of the public in aspects of health‐care planning, decision‐making, and priority‐setting.^1^ In addition, major reforms to the UK's National Health Service (NHS) have consistently invoked references to greater public involvement.^2^ In the USA, the recently created Patient‐Centered Outcomes Research Institute, an independent funding body endowed by the federal government, requires that all grantees provide an engagement plan detailing how investigators will involve patients and other stakeholders in their research.^3^

Given the enthusiasm for PPI‐based policies, there remains a surprising degree of uncertainty about PPI's philosophical foundations. Beyond general agreement that PPI refers to the involvement of laypeople in decisions related to health research, practice, or policymaking, there is little consensus on the precise meaning of PPI. Reading the vast literature on PPI, one encounters divergent assumptions about nearly every aspect of PPI: who it should involve, how it should be done, when it should be done, and so forth. Surveying the chaos, two recent commentators conclude that “PPI operates as an empty signifier, intermittently populated with whatever policy ideas of citizen engagement are *a la mode*.”^4^

Given the range of opinions on what PPI entails, there is also a great deal of confusion about the underlying justification, or justifications, for PPI. Some have maintained that there are different models of PPI that reflect distinct underlying ideological commitments.^5^ Others suggest that PPI reflects both moral and pragmatic motivations but that these motivations cannot be jointly realized, creating a troubling dilemma PPI practitioners.^6^ Ultimately, confusion about the normative underpinnings of PPI creates substantial obstacles for efforts to assess the success of PPI‐based policies. While there is a recognized need to evaluate and improve PPI‐based policies, most attempts to understand the strengths and weakness of different policies can be fairly described as descriptive rather than genuinely evaluative.^7^

It is beyond the scope of this article to address, let alone attempt to resolve, all the philosophical ambiguities that dog PPI‐based policies. However, we believe that an important step towards clarifying the meaning and justifications of PPI is to split apart the familiar acronym, drawing a distinction between patient and public involvement, rather than treating PPI as a single practice. Specifically, we will argue that the justification for involving the public differs in ethically significant ways from the justification for involving patients in decisions about health research or policy and that this difference can have important implications for what counts as success or effectiveness in any particular instance of involvement. Indeed, while the activities associated with patient involvement and public involvement may look similar on the surface, their underlying justifications are not just different, they are fundamentally in tension. Understanding the tension between reasons for involving patients and reasons for involving the public will enable policymakers and participants to be clearer about their intended goals in pursuing a PPI‐based policy and consequently, to be more precise in determining who to involve and how to involve them effectively.

## 2 PRIOR LITERATURE

Notwithstanding the continued practice of grouping patient and public involvement together under a common heading, there have been prior efforts to distinguish the two types of involvement. Florin and Dixon were among the first to claim straightforwardly that “public involvement is different from patient involvement.”^8^ Their account of the distinction turns less on differences between the characteristics of patients and the public than on differences between the domains in which the two forms of involvement occur. As they put it, public involvement refers to “the involvement of members of the public in strategic decisions about health services and policy at local or national level,” while patient involvement refers to “the involvement of individual patients, together with health professionals, in making decisions about their own health care.”^9^

This way of distinguishing between patient and public involvement is adopted, in large part, by Williamson, who couches the distinction in terms of private (i.e., patient) versus public participation.^10^ Williamson notes that “health policy can benefit from being informed by the private experience of citizens,” implying that there may be a role for private or patient involvement in public policy.^11^ However, she does not clarify when or for what reasons decision‐makers should seek private or patient rather than public input or how exactly the two types of input should be distinguished. Returning to the language of patient versus public involvement, Warsh criticizes Williamson for noting descriptively but failing to explore “conceptually and philosophically” the underlying justifications for patient and public involvement.^12^ However, he does not explicitly challenge the assumption that patient involvement takes place, at least primarily, in relation to decisions about one's own care.

There is, undoubtedly, an important distinction to be drawn between the practice of involving people in decisions about their own care and the practice of involving people in decisions about research or the broader health system. We agree with both Williamson and Florin and Dixon that these two forms of involvement ought not be conflated. However, the subtler but crucial distinction that these accounts fail to address directly is the one suggested by Williamson's reference to policy that is informed by private experiences. Specifically, in matters of research or public policy, when should decision‐makers seek input from patients drawing on their personal experience of a particular condition and when should they involve members of the general public? We believe that it is this patient/public distinction that tends to vex decision‐makers and for which greater clarity is required.

In perhaps the most thoroughgoing attempt to date to disentangle patient and public involvement, Fredriksson and Tritter make headway towards distinguishing between justifications for patient and public involvement in matters of research and public policy.^13^ First, they note that patients involved in health‐care decision‐making are typically expected to have vested interests related to their own care, whereas the public is expected to provide a “disinterested perspective.”^14^ In addition, they suggest that patients can bring experiential knowledge to decision‐making, whereas the public is not assumed to have special expertise when their views on health‐care‐related issues are solicited.^15^ These expectations about the characteristics of patients and the public should play a critical role in determining when and for what reasons different forms of involvement may be appropriate. Ultimately, however, Fredriksson and Tritter remain wedded to the domain‐based distinction introduced by Florin and Dixon, suggesting that “patient involvement focuses on people's participation in decisions about their own treatment and care or a group of patients helping to shape a particular service.”^16^ They add that patient involvement, conceived in this way, reflects “an effort to enhance autonomy” and resist “medical paternalism.”^17^

Thinking about patient involvement as a way to enhance patient autonomy and push back against medical paternalism makes sense if we assume that patient involvement primarily refers to patients becoming more active and empowered participants in decisions about their own care. However, the values of autonomy and anti‐paternalism cannot explain when, if ever, patients drawing on experiential knowledge, as opposed to members of the general public, should be involved in matters of research or policymaking, where decisions about their own care are not directly implicated. Thus, despite their keen insights into the different characteristics of patients and the public, Fredriksson and Tritter's analysis may be a somewhat confusing guide to researchers and policymakers trying to determine whom, if anyone, they should seek to involve in their decision‐making.

Finally, Trish Greenhalgh has recently proposed a set of terms and roles for laypersons involved in health research and policy that furthers efforts to distinguish patient and public involvement, while going beyond the simple patient/public dichotomy.^18^ One notable aspect of Greenhalgh's approach is that she does not tie distinctions between different types of lay involvement to the domains in which they take place but to the sorts of input that different categories of those involved can offer. She suggests that the term public is relevant when we want “accountability to the public”, citizen when we require “someone to represent the public” and lay person when we need “the voice of common sense.” These three categories of laypersons are distinct from another set of categories: patient, client, carer, service user, consumer and advocate. With respect to these latter categories, Greenhalgh suggests that the term patient is relevant when we seek “experts in experience”, carer when we refer to someone with “expertise in what it means to care for someone” and advocate when we are “seeking the perspective of an individual or group who finds it awkward or impossible to provide that perspective themselves.”^19^

Greenhalgh's terminology is useful for making fine‐grained distinctions among different types of laypersons involved in health policy and research. Importantly, however, the terms she proposes divide neatly into two broad categories that correspond to the characteristics of patients and the public distinguished by Fredriksson and Tritter. In particular, Greenhalgh's categories of patient and carer both explicitly reference the special expertise that these persons can contribute, while the category of advocate, though not explicitly tied to expertise, is associated with particular perspectives of individuals or groups. By contrast, the categories of the public, citizens, and laypersons are all associated with some notion of public or common views rather than special expertise.

In what follows, we build on these previous efforts by laying out a novel account of the distinction between patient and public involvement. Like the account of Fredriksson and Tritter, our account of the distinction is informed by the different characteristics of patients and the public. However, we reject the notion that patient involvement refers primarily to involvement in decisions about one's care or necessarily reflects an effort to enhance autonomy. Rather, we argue that the distinction between patient and public involvement turns on the different contributions that both groups can make to decision‐making, whether it relates to research, practice, or policy.

## 3 DEFINING KEY TERMS

Before exploring the distinct justifications for patient and public involvement it is necessary to clarify how we will use the terms going forward. We use the term patient involvement to refer to the practice of involving individuals in health research or policy on the basis of their experience of a particular disease or condition, including its effects on daily life and its management. We acknowledge that, used in this sense, the term patient involvement functions as a shorthand that obscures some relevant distinctions among individuals who have this sort of firsthand experience. For instance, some individuals who have or have had a particular illness or condition might refer to themselves not as patients but as consumers, service users, or survivors. Additionally, there are family members and other carers who do not have a particular illness or condition but nonetheless have direct experience of its effects on daily life. Thus, as we use the term here, patient involvement involves a degree of generalization. Nonetheless, we believe that the use of a single term highlights common characteristics of individuals involved in health research or policy on the basis of their firsthand experience with a particular illness or condition.

By contrast, we use the term public involvement to refer to the practice of involving individuals in health policy or research based on their status as members of a relevant population—be they residents of a city, individuals served by a health system, or a national population. Of course, many members of the public will also be patients or carers, but what matters in instances of public involvement is that they are involved irrespective of whether or not they happen to have firsthand experience of a particular illness or condition.

## 4 PATIENT INVOLVEMENT

In some cases, researchers, clinicians, or policymakers choose to consult patients or those connected to them because those patients can provide insights or expertise lacking in other parts of the research team or health‐care system. For example, researchers studying prostate cancer at the University College Hospital in London have enlisted the help of patients to try to take a holistic approach to the study of prostate cancer.^20^ By working with men who either have or have had prostate cancer, the researchers have identified certain quality of life factors related to prostate cancer and its treatment that might otherwise have gone overlooked. As part of the involvement process, researchers asked patients to identify the relative importance of factors like length of hospital stay, number of procedures required, and side‐effects of treatment. In addition, the men enlisted were able to help researchers understand how to reach out to men for the purposes of prostate cancer research, given the delicate nature of the disease.

Though the researchers could have theoretically drawn on any man in the population as since men serve as potential future patients, they chose to draw on patients who had the disease as the information they sought was informed by the experiences of individual patients. The reason, in other words, to involve patients in this case is *because they were patients*, who bring to the table special expertise informed by their experiences.

In the USA, the Food and Drug Administration (FDA) has pursued a multi‐year programme of patient involvement as part of its patient‐focused drug development initiative. Through a series of meetings with members of different patient populations, regulators at the FDA have sought a greater understanding of patients’ perspectives on “their disease, its impact on their daily life, and currently available therapies.”^21^ By consulting those with firsthand knowledge of what it is like to live with a particular condition and to experience the benefits and side‐effects of different therapies to treat that condition, FDA officials have sought to gain insights that might inform the risk‐benefit assessments they make in evaluating new therapies.

Similar evidence of the value of patient input can be found in the realm of the design of informed consent protocols and other areas of medical research.^22^ Across these settings, the underlying rationale for patient involvement is essentially the same: to tap into the unique insights and expertise of patients and those who are closely connected to them in order to improve research, care, or policy.

This is not to say that patient involvement has no other benefits. Some have suggested, for instance, that patient involvement in research has the potential to address power imbalances between researchers and patients, helping patients to feel a greater sense of empowerment and encouraging researchers to view patients as potential partners rather than passive research subjects.^23^ Note, however, that these benefits depend to a significant degree on it being the case that patients are in fact able to make valuable contributions to planning or conducting research. Involving patients in aspects of research that they are not equipped to contribute to is likely to frustrate and disillusion rather than empower them.^24^ Likewise, patient involvement is not likely to encourage researchers to rethink their views of patients if their involvement does not actually add value to research. Thus, while these additional benefits of patient involvement may be substantial, they should be considered ancillary to the primary goal of patient involvement, which is to improve research or policy.

## 5 PUBLIC INVOLVEMENT

The primary rationale for involving members of the general public in decisions about research or policy is to bring a level of impartiality or independence to debates about a particular reform, policy, or governance or funding decision with wider implications for the health system. Debates of this sort are, of course, often dominated by stakeholders, including physicians, health‐care providers, industry representatives, patient groups, and affected patients, all of whose views may be biased by their vested interests. To counterbalance the views of these different stakeholder groups, decision‐makers sometimes seek the input of individuals who do not hold a particularly strong individual interest in the issue being discussed and who, therefore, can function in the role of neutral arbiters.^25^ Much like the individuals summoned for a criminal jury, individuals involved in public involvement activities come from diverse backgrounds, but ideally come without an established position on the issue at hand and with a willingness to consider competing perspectives.

Understood in these terms, public involvement has limited applications in clinical settings, and clearer applications in policy settings where decisions about issues such as the distribution of scarce resources or the redesign of health‐care systems require trade‐offs between competing values and interests. In these contexts, public involvement activities are substantially informed by theories of deliberative democracy, which emphasize that the considered normative opinions of the lay public should be given “due weight in policy making and implementation.”^26^ The connection to deliberative democracy also helps to clarify the of value of public involvement. Whereas patient involvement is justified primarily on the basis of its instrumental value to research or policymaking, the value of public involvement is primarily procedural. It can form part of a fair process for making decisions that pit different interests against each other by helping to ensure that those decisions are informed by the considered opinions of a range of deliberators rather than dominated by the most organized or active stakeholder groups.

One method of public involvement, the citizens’ jury, has been used in efforts to develop criteria in priority setting for health technology assessment in Canada^27^ and setting priorities for health research in the UK.^28^ In these cases, jurors who are selected from a geographically defined population convene to hear testimony from expert witnesses and to deliberate with each other before rendering a decision or recommendation on the issue at hand.^29^ While citizens’ juries are typically organized on an ad hoc basis to address particular policy challenges as they arise, other approaches to public involvement, like the Citizens Council of the National Institute for Health and Clinical Excellence (NICE), rely on standing bodies of citizen deliberators. The Council is composed of 30 members recruited to reflect the demographic make‐up of England and Wales. Council members serve staggered 3‐year terms and meet twice a year for 3 days to discuss various issues on which NICE seeks guidance.^30^ In the years since its inception, the Council has addressed issues including health inequalities, clinical need, and the rule of rescue.^31^ As former NICE chairman Michael Rawlins put it, the Council is intended “to reflect the attitudes of the general public rather than those with professional knowledge and experience of healthcare and the NHS.”^32^ For this reason, individuals employed in the NHS, private medicine, health‐care industries, or patient advocacy groups are excluded from participation in the Council. Acting as an impartial deliberative body, the Council is designed to ensure that when making social value judgments, NICE's decision‐makers take account of a broad range of views rather than imposing personal prejudices.^33^

By contrast with patient involvement, wherein individual are selected based on their close connection to a particular condition, it is the involved individuals’ lack of a personal connection to the issue at hand that justifies their inclusion in public involvement activities. Of course, it would be naive to assume that any individual can come to the table without any strongly held beliefs or ancillary interest. Organizers of public involvement activities try to minimize these biases by convening diverse groups of people, so that even if each individual's perspective reflects her particular circumstances, a group of involved individuals is not characterized by systematic biases. Additionally, as noted, public involvement activities tend to utilize deliberative decision‐making procedures that encourage individuals who are involved to consider other viewpoints and to provide justifications for their own opinions. Even with deliberative procedures in place, the key assumption behind the decision to involve the general public rather than patients is that individuals are more likely to be able to look beyond their self‐interest when their personal background does not heavily bias them in one direction or another.

## 6 BENEFITS OF THE DISTINCTION

Distinguishing between patient and public involvement in the terms we propose has both theoretical and practical benefits. One theoretical benefit of the distinction is that it dissolves a supposed paradox at the heart of PPI in applied health research. Diagnosed by Ives et al., this paradox is said to stem from the fact that there are two distinct rationales for PPI that cannot be fully reconciled in practice.^34^ The first rationale, which is “pragmatic” and “outcome‐oriented”, is that “patients and the public are said to bring unique perspectives to research, making it more appropriate to their needs and the needs of healthcare services.”^35^ The second rationale, which is “rights based” and “process oriented” is said to be rooted in “broader social and ethical narratives around democratic representation, transparency, accountability, responsibility and the redressing of power imbalances.”^36^

Ives et al. suggest that realizing these different rationales requires different approaches to PPI. The pragmatic rationale for PPI requires only a transactional relationship between the persons involved and researchers, where the former provide insight and expertise as appropriate but are not required to have any role in decision‐making.^37^ By contrast, achieving the process‐oriented rationale for PPI requires a cooperative approach wherein involved persons do not simply provide evidence to inform decisions but actually participate in decision‐making itself. The paradox‐generating problem, Ives et al. suggest, is that “unless PPI agents obtain adequate training, they cannot contribute substantially to the conduct of research” in the manner required to realize the more robust process‐oriented rationale for PPI.^38^ However, “once a PPI agent undergoes training, and becomes familiar enough with research to be substantially involved, their ‘lay’ status is compromised.”^39^

Our proposed distinction reveals this supposed paradox to be an artefact of lumping together two distinct forms of involvement with two distinct rationales under the common heading of PPI. On our account, what Ives et al. refer to as the pragmatic and outcome‐oriented rationale for PPI—namely, that involved persons “bring unique perspectives to research, making it more appropriate to their needs and the needs of healthcare services”—is the rationale for *patient* involvement. What they refer to as the process‐oriented rationale for PPI—namely, that involved persons promote “democratic representation, transparency, accountability”—is the rationale for *public* involvement. Once we abandon the notion that PPI refers to a single practice, the assumption that these two rationales need to be reconciled falls away.

Nonetheless, it might look as though simply distinguishing between patient and public involvement fails to address all of Ives et al.'s concerns. If we assume that realizing the goals of *public* involvement still requires giving involved persons a cooperative rather than a merely consultative role in decision‐making, it might seem to be the case that training lay persons for this cooperative role would compromise their lay status. But as we've suggested above, another benefit of distinguishing between patient and public involvement is that it clarifies the fact that the two types of involvement are suited to different types of decisions. There is no reason to think that members of the general public would have special insights into the subjective experience of particular conditions or the benefits and burdens associated with different treatments. Thus, there is no reason to think that they should be involved in decisions about the design of clinical trials or other technical issues with respect to which they bear no special expertise. However, when it comes to high‐level decisions that involve weighing costs and benefits of different policies or lines of research, the experience of NICE's Citizens’ Council and numerous citizens’ juries suggests that members of the public can be educated to a sufficient degree to understand the tradeoffs at stake and to deliberate about the common good without undergoing training so transformative as to compromise their lay status.

A key practical benefit of introducing a clear distinction between patient and public involvement is that it gives researchers a framework for structuring complex involvement activities that combine elements of both patient and public involvement. The Assessing Service and Technology Use to Enhance Health (ASTUTE) health study, which was established in Australia in 2009, provides one example of how the distinction might guide practice.^40^ This study was concerned with the benefits and feasibility of disinvestment seeking to steer health system resources from unsafe or ineffective health services to those with better safety or effectiveness profiles. A principal aim of the study was “to determine whether stakeholder engagement could contribute to creating improved and transparent decision‐making processes in this policy domain.”^41^ One of the first cases selected for the study dealt with was assisted reproductive technologies (ART), specifically in vitro fertilization and intracytoplasmic sperm injection, both of which were subsidized under Australia's universal health‐care system.

As part of their stakeholder engagement efforts, investigators recruited both women who had undertaken ART—a clear instance of patient involvement, according to our definition—as well a group of persons “matched against predetermined stratification criteria for broadly proportional representation of the Australian population” none of whom had experience with ART—an instance of public involvement, according to our definition.^42^ Although the ASTUTE investigators did not distinguish between patient and public involvement in the precise terms that we propose, the structure of the study evinced their implicit understanding of the distinction. In particular, the investigators acknowledged important differences between individuals who had undertaken ART and those who had been selected for demographic representation. They referred to the former group as partisan or vested stakeholders and the latter as non‐partisan community members, convening the two groups of lay individuals in separate deliberative sessions.

While this categorization of different stakeholder groups shows an implicit awareness of the distinct characteristics of patients and members of the general public, a more thorough understanding of the unique underlying rationales for patient and public involvement might have improved this study further by clarifying the value of each type of involvement. The ASTUTE investigators hypothesize “that deliberative engagement with the community will be seen to add valuable information to a policy decision, helping to balance the vested interests of single‐issue consumer groups.”^43^ However, they add “it may be that the outcomes of engagement with partisan stakeholders are viewed with more caution.”^44^ The distinction we defend here explains why this would be the case. Public involvement has value, as the authors suggest, precisely because it can bring an impartial perspective to an issue that is dominated by vested interests. Thus, observers would be right to view the involvement of partisan stakeholders as antithetical to such efforts. Similarly, though the investigators suggest that both community members and vested stakeholders might contribute colloquial evidence to decision‐making about ART, it seems far more likely that the vested stakeholders would have such evidence to offer, precisely because they have firsthand experience of the technologies being discussed.

As this example shows, even though instances of public and patient engagement may be combined as part of a broader programme of lay involvement, a clear understanding of the rationale for each can help decision‐makers structure the involvement activities in a way that reflects the aims of each sort of involvement.

## 7 GREY AREAS

One possible objection to the distinction we have laid out is that it is too neat and that there are cases of lay involvement that fall into a grey area between our proposed categories. One type of lay involvement that may appear to challenge the distinction is the community consultation required in the USA for research conducted in emergency settings using an exemption from informed consent (EFIC). Guidance from the FDA states that community consultations should include representatives of both “the community in which the research will be conducted,” meaning the geographical area where the hospital or study site is located and “the community from which subjects will be drawn,” defined as the “group of patients who share a particular medical or other characteristic that increases the likelihood that they (or a family member) may be enrolled in the study,” a group may that also include patients with firsthand experience of the disease or condition being studied.^45^ Do these community consultations, which aim to involve persons from both the geographical community and the condition community (as they are sometimes called),^46^ show that in at least some cases, it doesn't make sense to distinguish between patient and public involvement?

Several comments are in order. First, it bears noting that the FDA offers a range of rationales for the community consultations, which, as summarized by Scicluna et al. include “expressing respect, promoting trust within the community, providing opportunities to suggest improvements or alterations to the design or conduct of the study, and helping to engage the community in research efforts more generally.”^47^ One possibility is that achieving these different rationales requires involving different groups of individuals. For instance, it is plausible that if researchers want to hear suggestions for improvements to the study design they would be better off talking to those with a close connection to the condition being studied. By contrast, if the goal is to show respect for and promote trust within the larger community, this might be accomplished by having a diverse group of members of the geographical community weigh in on the balance of harms and benefits entailed by the proposed study. In this way, if the different rationales for community consultation were disaggregated and specified, it might turn out to be the case that the corresponding elements of patient and public involvement could be separated as they were in the ASTUTE study.

For the sake of argument, though, let us assume that the community consultations for EFIC research cannot or shouldn't be broken into discrete public involvement and patient involvement sessions. Even in this case, the distinction between patient and public involvement can help to highlight the tradeoffs at stake in prioritizing the inclusion of the geographical community over the condition community or vice versa. Research has shown that individuals with personal experience of a particular condition are more supportive of EFIC research addressing that condition than those without such experience.^48^ At the same time, it is reasonable to assume that individuals with experience of the condition might also have more informed opinions about research to treat that condition. Thus, by prioritizing the inclusion of people with a personal connection to the disease, sponsors of a community consultation might increase the likelihood that the session will yield insightful input that can be used to improve the trial design. However, they would open themselves up to the justifiable criticism that they had not listened to the full range of concerns from individuals living in proximity to the study site who might be enrolled in the trial. By contrast, consultation sponsors might prioritize involving a diverse range of individuals living near the trial site in order to capture a wide array of views about the risks and benefits of the research, but to the extent that these individuals have no firsthand experience of the disease being studied, this would decrease the likelihood that the consultation would yield valuable suggestions for the modification of the trial design. Thus, the fact that there are examples like community consultation which apparently combine patient and public involvement does not obviate the distinction we have argued for here. On the contrary, in these hybrid cases, bearing in mind the distinct rationales for patient and public involvement can help to highlight tradeoffs between different strategies and goals of involvement that might otherwise go unnoticed.

## 8 CONCLUSION

There is little question that the direction of the health‐care system is towards greater lay involvement in decision‐making. Amid this transition, we argue that overarching homogeneous PPI policies which pay little attention to the different reasons for involving patients and the public do little to help researchers, policy‐makers, patients, and members of the general public collaborate in more meaningful ways and can lead to misunderstandings with implications for trust and confidence in health‐related decision‐making. Though not mutually exclusive, the motivations to involve patients or the general public in decisions about research, policy, and practice are fundamentally different. Going forward, policies that evince an understanding of these differences will better serve the interests of researchers, policy‐makers, patients, and the public.

## CONFLICT OF INTEREST

The author declares no conflict of interest.

## Footnotes

1
Health and Social Care Act (2001). Retrieved from http://www.legislation.gov.uk/ukpga/2001/15/contents (Accessed 8 November 2018).

2
Daykin, N., Evans, D., Petsoulas, C., & Sayers, A. (2007). Evaluating the impact of patient and public involvement initiatives on UK health services: a systematic review. *Evidence & Policy*,* 3*(1); Staniszewska, S., Herron‐Marx, S., & Mockford, C. (2008). Measuring the impact of patient and public involvement: the need for an evidence base. *International Journal for Quality in Health Care*,* 20*(6): 373–74; Staniszewska, S., Adebajo, A., Barber, R., Louca, P. B., Brady, M., Brett, J. … Williamson, J. (2011). Developing the evidence base of patient and public involvement in health and social care research: the case for measuring impact. *International Journal of Consumer Studies, 35*(6), 628–632.

3
Patient‐Centered Outcomes Research Institute. (2014). *The value of engagement*. 12 August. Retrieved from https://www.pcori.org/engagement/what-we-mean-engagement.

4
Madden, M., & Speed, E. (2017). Beware zombies and unicorns: Toward critical patient and public involvement in health research in a neoliberal context. *Frontiers in Sociology,* 2, 5. doi: /10.3389/fsoc.2017.00007.

5
Forbat, L., Hubbard, G., & Kearney, N. (2009). Patient and public involvement: Models and muddles. *Journal of Clinical Nursing*,* 18*(18), 2547–2554.10.1111/j.1365-2702.2008.02519.x19207798

6
Ives, J., Damery, S., & Redwod, S. (2013). PPI, paradoxes and Plato: Who's sailing the ship? *Journal of Medical Ethics, 39*(3), 181–185.10.1136/medethics-2011-10015022267385

7
Op cit. note 4, p. 2.

8
Florin, D., & Dixon, J. (2004). Public involvement in health care. *BMJ 328*(7432), 159–161, p. 159.10.1136/bmj.328.7432.159PMC31451914726350

9
Ibid.

10
Williamson, L. (2014). Patient and citizen participation in health: The need for improved ethical support. *American Journal of Bioethics, 14*(6), 4–16.10.1080/15265161.2014.900139PMC416028424809598

11
Ibid., p. 5.

12
Warsh, J. (2014). PPI: Understanding the difference between patient and public involvement. *American Journal of Bioethics, 14*(6), 25–26, p. 26.10.1080/15265161.2014.90014424809603

13
Fredriksson, M., & Tritter, J. Q. (2017). Disentangling patient and public involvement in healthcare decisions: why the difference matters. *Sociology of Health & Illness, 39(*1), 95–111.10.1111/1467-9566.1248327862007

14
Ibid., p. 100.

15
Ibid.

16
Ibid., p. 99.

17
Ibid., p. 98.

18
Greenhalgh, T. (2017). *How to implement evidence‐based healthcare*. Hoboken, NJ: Wiley‐Blackwell, p. 100.

19
Ibid.

20
University College London Joint Research Office (n.d.). How patients have helped us with our research. UCLH/UCL NIHR Comprehensive Biomedical Research Centre.

21
Food and Drug Administration. (n.d., last updated 2018). *The voice of the patient: A series of reports from FDA's patient‐focused drug development initiative*. Available at: https://www.fda.gov/ForIndustry/UserFees/PrescriptionDrugUserFee/ucm368342.htm Accessed 30 June 2017.

22
Guarino, P., Elbourne, D., Carpenter, J., & Peduzzi, P. (2006). Consumer involvement in consent document development: a multicenter cluster randomized trial to assess study participants’ understanding. *Clinical Trials*,* 3*(1), 19–30; Boote, J. Telford, R., & Cooper, C. (2002). Consumer involvement in health research: a review and research agenda. *Health Policy*,* 61*(2), 213–236.10.1191/1740774506cn133oa16539087

23
Green, G. (2016). Power to the people: To what extent has public involvement in applied health research achieved this? *Research Involvement and Engagaement*,* 2*(1), 1–13.10.1186/s40900-016-0042-yPMC583188829507763

24
Domecq, J. P., Prutsky, G., Elraiyah, T., Wang, Z., Nabhan, M., Shippee, N., … Murad, M. H. (2014). Patient engagement in research: a systematic review. *BMC Health Serv Res 14*, 89.10.1186/1472-6963-14-89PMC393890124568690

25
Parkinson, J. (2004). Hearing voices: Negotiating representation claims in public deliberation. *British Journal of Politics and International Relations*,* 6*(3), 370–388: 385.

26
Kim, S. Y. H., Wall, I. F., Stanczyk, A., & De Vries, R. (2009). Assessing the public's views in research ethics controversies: Deliberative democracy and bioethics as natural allies. *Journal of Empirical Research on Human Research Ethics, 4*(4), 3–16, p. 3.10.1525/jer.2009.4.4.3PMC285379719919315

27
Menon, D., & Stafinski, T. (2008). Engaging the public in priority‐setting for health technology assessment: Findings from a citizens’ jury. *Health Expectations*;* 11*(3), 282–293.10.1111/j.1369-7625.2008.00501.xPMC506045118816324

28
Gooberman‐Hill, R. Horwood, J., & Calnan, M. (2008). Citizens’ juries in planning research priorities: process, engagement and outcome. *Health Expectations*,* 11*(3), 272–281.10.1111/j.1369-7625.2008.00502.xPMC506044918816323

29
Smith, G., & C. Wales, C. (1999). The theory and practice of citizens’ juries. *Policy & Politics, 27*(3), 295–308.

30
Rawlins, M. (2009). Background to NICE's citizens council. In P. Littlejohns & M. Rawlins (Eds.). *Patients, the public and priorities in healthcare*. London: Radcliffe.

31
Littlejohns, P. (2009). The Citizens Council reports. In Littlejohns, & Rawlins, op cit. note 30.

32
Op cit. note 30.

33
Ibid.

34
Ives *et al*., op cit. note 6.

35
Ibid., p. 181.

36
Ibid., p. 182.

37
Ibid.

38
Ibid., p. 183.

39
Ibid.

40
Watt, A. M., Hiller, J. E., Braunack‐Mayer, A. J., Moss, J. R., Buchan, H., Wale, J. … Elshaug, A. G. (2012). The ASTUTE Health study protocol: Deliberative stakeholder engagements to inform implementation approaches to healthcare disinvestment. *Implementation Science,* 7(1), 101.10.1186/1748-5908-7-101PMC352086323088222

41
Ibid., p. 2.

42
Ibid., p. 6.

43
Ibid., p. 11.

44
Ibid.

45
Guidance for Institutional Review Boards, Clinical Investigators, and Sponsors: Exception from Informed Consent Requirements for Emergency Research. (2013). US Food and Drug Administration: 25.

46
Scicluna, V. M., Ali, M. K.,. Pentz, R. D., Wright, D. W., & Dickert, N. W. (2017). Does experience matter? Implications for community consultation for research in emergency settings. *AJOB Empirical Bioethics*,* 8*(2), 75–81.10.1080/23294515.2017.130897828949843

47
Ibid., p. 75.

48
Op cit. note 46.

